# Hemorrhoidal Artery Ligation Operations-Recto-Anal Repair (HALO-RAR) Procedure for Recurrent Haemorrhoids: Excellent Patient Satisfaction

**DOI:** 10.7759/cureus.7944

**Published:** 2020-05-03

**Authors:** Mohammad Miah, Daniel Centea, Gabriel Michael, Najam Husain, Ioannis Virlos, Mohamad Al Saramigy

**Affiliations:** 1 General Surgery, University Hospitals of Derby and Burton National Health Service (NHS) Foundation Trust, Burton on Trent, GBR; 2 General and Colorectal Surgery, University Hospitals of Derby and Burton National Health Service (NHS) Foundation Trust, Burton on Trent, GBR

**Keywords:** halo (haemorrhoidal artery ligation operation), doppler-guided, proctoscopy, rar (recto-anal repair)

## Abstract

Background

Haemorrhoid is the most common anal canal disease. Treatments may vary from non-invasive to invasive depending on the symptoms. Haemorrhoidectomy has been widely used. However, it has some drawbacks like severe postoperative pain, longer time to return to daily activities and complications such as anal stenosis. To overcome these, various new treatment methods have been introduced. Doppler-guided hemorrhoidal artery ligation operations (HALO) are becoming popular among surgeons. HALO has been reported to have a lower recurrence rate of less than 10% and higher patient satisfaction of approximately 90% with minimal postoperative pain. It achieves very good postoperative outcomes in the treatment of early haemorrhoids where per rectal bleeding and/or perianal discomfort are main symptoms. Nevertheless, it has a limitation in the treatment of prolapsing haemorrhoids. To tackle this, simultaneous recto-anal repair (RAR) has been recently introduced. HALO, in combination with RAR, has been reported to achieve good postoperative outcomes and excellent patient satisfaction.

This is a two-stage open operation. The stages are:

- Doppler-guided HALO and

- RAR (recto-anal repair)

Methods

We are presenting a single-centre one-year experience of Doppler-guided haemorrhoidal artery ligation operation and recto-anal repair (DG-HALO and RAR) conducted on haemorrhoidal patients to evaluate the outcomes and effectiveness of the procedure. Retrospective data were collected for the patients who underwent HALO over one year period from June 2018 to August 2019. A total of 10 patients were treated with the HALO-RAR procedure.

Results

The male to female ratio was 7:3, median age was 47.98 (28.38 - 61.7) years, median body mass index (BMI) was 30.23 (23.8 - 39.1). Eight patients were American Society of Anesthesiologists (ASA) Grade II, one patient was ASA I and one was ASA III. Time from initial consultation to the HALO procedure was 9.90 (3.5 - 19.8) months. All patients complained of preoperative bleeding and six of them complained of pain or discomfort. Nine patients underwent previous bandings in the clinic and one patient declined banding. The average time of the procedure was 57 mins. The average number of ligations was 10 (0-21). In one case, the proctoscope did not pair with the speaker. The average number of plications was three (2-4). Postoperatively, nine patients had no immediate complications; one patient had acute urinary retention. Seven patients were discharged on the same day. One patient had to stay overnight for monitoring prior to restarting apixaban, one patient for his learning difficulties and one patient had an unplanned overnight stay due to acute urinary retention requiring catheterization. Eight patients had their first follow-up; improvement of symptoms was found in 100% patients on the first follow-up.

Conclusion

HALO-RAR should be considered as a treatment option for recurrent symptoms after banding for haemorrhoids. The study showed good overall results with no immediate surgical complications. Excellent patient satisfaction was found even in long-term follow-up.

## Introduction

A haemorrhoid is the most common anal canal disease. Treatments may vary from non-invasive to invasive depending on the symptoms. Non-invasive treatments include banding, cryotherapy and sclerotherapy. Invasive treatments include haemorrhoidectomy, which is widely used. However, it has some drawbacks like severe postoperative pain, longer time to return to daily activities and complications such as anal stenosis. To overcome these, various new treatment methods have been introduced. Doppler-guided hemorrhoidal artery ligation operations (HALO) are becoming popular among surgeons. HALO has been reported to have a lower recurrence rate of less than 10% and higher patient satisfaction of approximately 90% with minimal postoperative pain. It achieves very good postoperative outcomes in the treatment of early haemorrhoids where per rectal bleeding and/or perianal discomfort are the main symptoms. Nevertheless, it has a limitation in the treatment of prolapsing haemorrhoids. To tackle this, simultaneous recto-anal repair (RAR) has been recently introduced. HALO, in combination with RAR, has been reported to achieve good postoperative outcomes and excellent patient satisfaction. 

HALO-RAR procedure

This is a two-stage open operation. It treats both internal and external haemorrhoids that are bleeding and prolapsing without complete removal of the haemorrhoidal tissue. The stages are:

 - Doppler-guided HALO (haemorrhoidal artery ligation operation), which stops the blood supply to the haemorrhoidal cushion by ligating the specific haemorrhoidal artery and

 - RAR (recto-anal repair), which fixes the prolapsing part of the haemorrhoid back into its normal place in the rectum by plicating stitches

HALO involves introducing a proctoscope with an attached micro-Doppler probe in the anus. It identifies the haemorrhoidal artery proximal to the haemorrhoidal tissue. The vessel is then ligated through a gap (window) in that same area on the proctoscope.

During RAR, the Doppler probe is removed from the anal canal. Plication sutures are then placed along the length of the prolapsed haemorrhoidal cushion. Finally, these running stitches are then tightened to draw up the prolapsed haemorrhoidal tissue.

## Materials and methods

We are presenting a single-centre, one-year experience of a Doppler-guided haemorrhoidal artery ligation operation and recto-anal repair (DG-HALO and RAR) conducted on haemorrhoidal patients to evaluate the outcomes and effectiveness of the procedure. It is a retrospective cohort study. Retrospective data were collected for the patients who underwent HALO over a one-year period from June 2018 to August 2019. A total of 10 patients were treated with the HALO-RAR procedure.

Patients who had previous bandings for their haemorrhoids with a recurrence of symptoms were included. All the new diagnoses of haemorrhoids were excluded.

## Results

**Figure 1:**
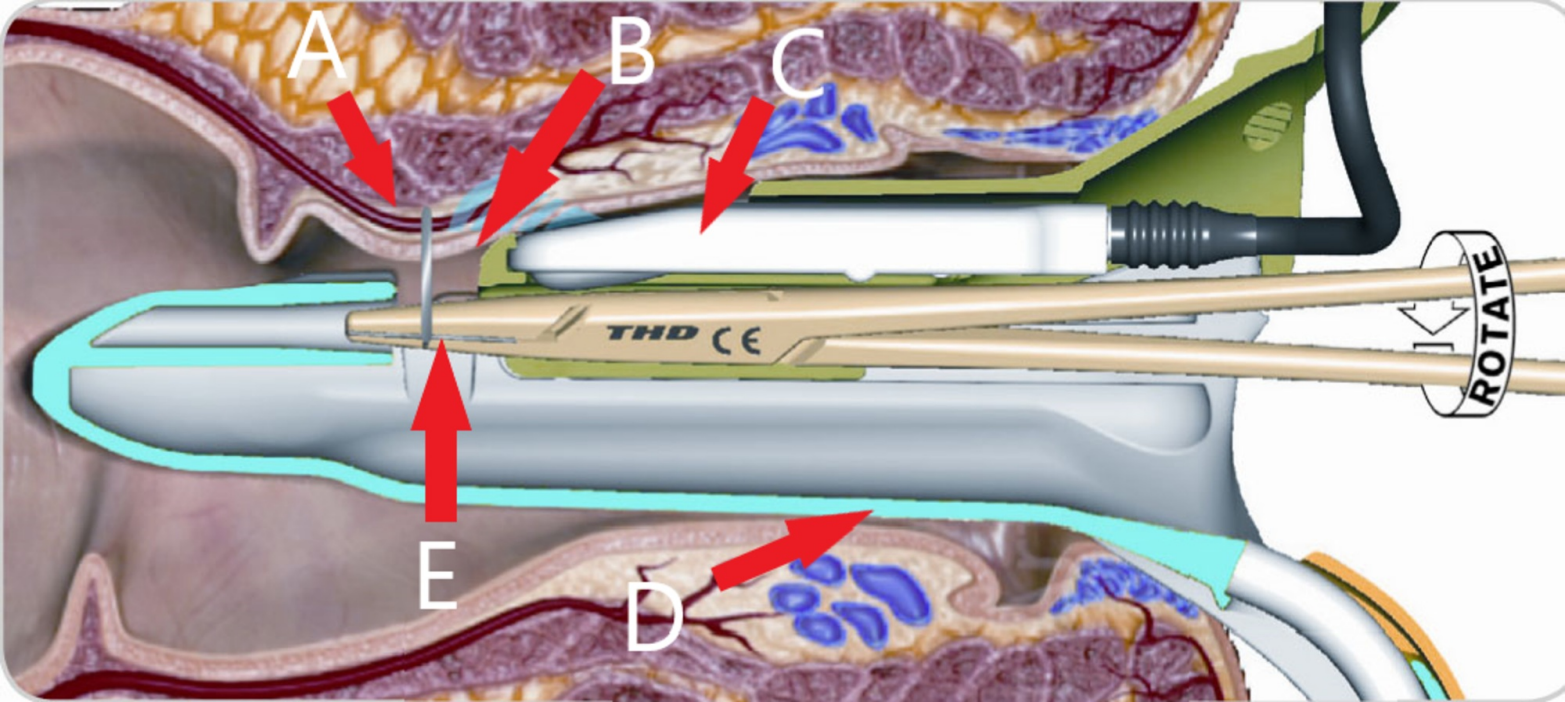
HALO part of the procedure showing the proctoscope with an embedded micro-Doppler ultrasound probe in the anus and ligation of the haemorrhoidal artery through a window on the proctoscope A- Haemorrhoidal artery; B- Window; C- Doppler probe; D- Proctoscope; E- Needle Holder HALO: Hemorrhoidal artery ligation operation

**Figure 2:**
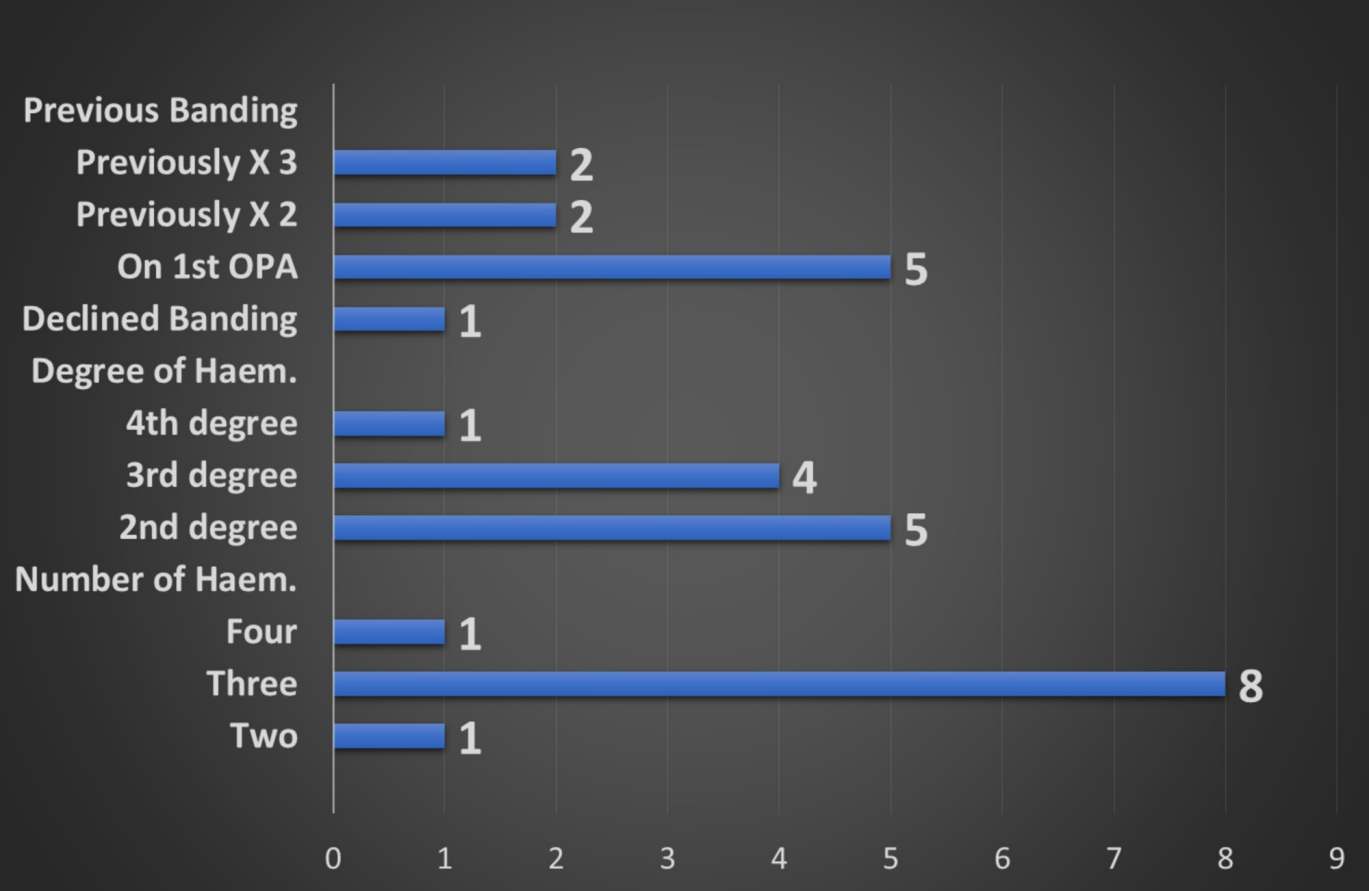
Showing the number of patients having previous bandings as well as the number and degree of haemorrhoids

The male-to-female ratio was 7:3, median age was 47.98 (28.38 - 61.7) years, median body mass index (BMI) was 30.23 (23.8 - 39.1). Eight patients were American Society of Anesthesiologists (ASA) Grade II, one patient was ASA I and one was ASA III. Time from initial consultation to the HALO procedure (Figure 1) was 9.90 (3.5 - 19.8) months. All patients complained of preoperative bleeding and six of them complained of pain or discomfort. Nine patients underwent previous bandings in the clinic and one patient declined banding (Figure 2). The average time of the procedure was 57 mins. The average number of ligations was 10 (0-21). In one case, the proctoscope did not pair with the speaker. The average number of plications was three (2-4) (Table 1). Postoperatively, nine patients had no immediate complications while one patient had acute urinary retention. Seven patients were discharged on the same day. One patient had to stay overnight for monitoring prior to restarting apixaban, one patient for his learning difficulties and one patient had an unplanned overnight stay due to acute urinary retention that required catheterization. Eight patients had their first follow-up. Improvement of symptoms was found in 100% of patients on the first follow-up. Seven out of eight patients reported satisfaction (the unsatisfied patient had 50% symptomatic improvement). However, there was variability in symptom resolution among the patients. Five patients had a second follow-up. One-hundred per cent patients (5 out of 5) were satisfied at the second follow-up; one patient did not arrive. Four out of five patients had complete resolution of symptoms. In one out of five, 80% improvement of symptoms was seen (previously 70%). One out of five had a 70% improvement in symptoms (previously 50%) - this patient who was not satisfied at the first follow-up was satisfied at the second follow-up. On examination, two out of five had skin tags. Three patients had a third follow-up (3 patients + 1 repeatedly did not arrive so he was discharged). One out of three patients had a complete resolution of symptoms. One out of three had a 90% improvement of symptoms (previously 80%). One out of three patients had a 70% improvement of symptoms (previously 60%). On examination, one patient had an anal skin tag and had a fourth follow-up. He mentioned a 70% improvement in his symptoms (previously 70%), however, intermittent bleeding and soiling were reported. 

**Table 1:** Showing the numbers of different procedures performed

Number of Ligations Performed	Patients	Number of Rectopexy Performed	Patients
11	3	4	4
12	2	3	4
8	2	2	2
14	1		
21	1		
0	1		

## Discussion

Haemorrhoids occur due to the engorgement of the vascular anal cushions. The symptoms may vary from bleeding, itching or perianal discomfort (Grade I) to large prolapsing haemorrhoids (Grades II-IV). Haemorrhoids that are prolapsed may reduce spontaneously after defaecation (Grade II); they may need manual re-positioning (Grade III) or they may be irreducible and remain continually prolapsed (Grade IV). Both Grades I and II haemorrhoids may be treated by dietary modification and topical cream or ointment application. Invasive treatments may include banding and sclerotherapy. Treatments for Grades III and IV haemorrhoids may require surgical excision of the haemorrhoidal tissue (open haemorrhoidectomy) or stapled haemorrhoidopexy. Doppler-guided HALO cuts off the blood flow to haemorrhoids, with the aim of reducing discomfort and bleeding by the shrinkage of the haemorrhoidal cushion due to a lack of blood supply [1]. HALO aims to achieve the downsizing of haemorrhoidal tissue but additional treatment is necessary for large prolapsing haemorrhoids. A case series of 616 patients managed by the HALO procedure without Doppler guidance reported symptom resolution at the four-week follow-up in 96%, 98% and 96% of patients who had presented with bleeding, prolapse and pain on defecation, respectively. In the same study, among 523 patients with a one-year follow-up, the mean patient satisfaction score was 8.2 on a 10-point scale [2]. In another case series of 330 patients, they reported resolution of symptoms at a mean follow-up of 46 months in 93% of patients who presented with bleeding and 92% of patients who presented with prolapsed haemorrhoids. In our study, improvement of symptoms was found in 100% patients on the first follow-up. Seven out of eight patients reported satisfaction (the unsatisfied patient had 50% symptomatic improvement). However, there was variability in symptom resolution among the patients.

## Conclusions

Finally, it is evident that a Doppler-guided HALO-RAR procedure can be considered a feasible treatment option in those patients with recurrent symptoms after banding for prolapsing haemorrhoids. The study showed good overall results with no immediate surgical complications. Excellent patient satisfaction was found even after four weeks. Improvement in symptoms in long-term follow-up was >80% with a very small impact on resource utilization. On top of these, it is a day case procedure with a short operative time. For better evidence, we recommend a larger sample size and a long-term follow-up. A telephonic follow-up (needs structured questionnaire) can be considered an alternative valid option.
